# KHK, PNPLA3 and PPAR as Novel Targets for the Anti-Steatotic Action of Bempedoic Acid

**DOI:** 10.3390/biomedicines10071517

**Published:** 2022-06-27

**Authors:** Ana Magdalena Velázquez, Roger Bentanachs, Aleix Sala-Vila, Iolanda Lázaro, Jose Rodríguez-Morató, Rosa María Sánchez, Juan Carlos Laguna, Núria Roglans, Marta Alegret

**Affiliations:** 1 Department of Pharmacology, Toxicology and Therapeutic Chemistry, School of Pharmacy and Food Science, University of Barcelona, Av. Joan XXIII 27–31, 08028 Barcelona, Spain; avelazquezpy@gmail.com (A.M.V.); bentanachs@ub.edu (R.B.); rmsanchez@ub.edu (R.M.S.); jclagunae@ub.edu (J.C.L.); 2 Cardiovascular Risk and Nutrition, Hospital del Mar Medical Research Institute (IMIM), 08003 Barcelona, Spain; asala3@imim.es (A.S.-V.); iolan.lazaro@gmail.com (I.L.); 3 Integrative Pharmacology and Systems Neuroscience Research Group, Hospital del Mar Medical Research Institute (IMIM), Dr. Aiguader 88, 08003 Barcelona, Spain; jose.rodriguez@upf.edu; 4 Department of Medicine and Life Sciences, Universitat Pompeu Fabra, Dr. Aiguader 88, 08003 Barcelona, Spain; 5 Spanish Biomedical Research Centre in Physiopathology of Obesity and Nutrition (CIBEROBN), Instituto de Salud Carlos III (ISCIII), 28029 Madrid, Spain; 6 Institute of Biomedicine, University of Barcelona, 08028 Barcelona, Spain

**Keywords:** NAFLD, triglycerides, fructose, bempedoic acid, fructokinase, β-oxidation

## Abstract

Bempedoic acid (BemA) is an ATP-citrate lyase (ACLY) inhibitor used to treat hypercholesterolemia. We studied the anti-steatotic effect of BemA, and the mechanisms involved, in a model of fatty liver in female rats obtained through the administration of a high-fat diet supplemented with liquid fructose (HFHFr) for three months. In the third month, a group of rats was treated with BemA (30 mg/kg/day) by gavage. Plasma analytes, liver histology, adiposity, and the expression of key genes controlling fatty acid metabolism were determined, and PPAR agonism was explored by using luciferase reporter assays. Our results showed that, compared to HFHFr, BemA-treated rats exhibited lower body weight, higher liver/body weight, and reduced hepatic steatosis. In addition to ACLY inhibition, we found three novel mechanisms that could account for the anti-steatotic effect: (1) reduction of liver ketohexokinase, leading to lower fructose intake and reduced de novo lipogenesis; (2) increased expression of patatin-like phospholipase domain-containing protein 3, a protein related to the export of liver triglycerides to blood; and (3) PPARα agonist activity, leading to increased hepatic fatty acid β-oxidation. In conclusion, BemA may represent a novel approach to treat hepatic steatosis, and therefore to avoid progression to advanced stages of non-alcoholic fatty liver disease.

## 1. Introduction

Bempedoic acid (BemA, 8-Hydroxy-2,2,14,14-tetramethylpentadecanedioic acid) [1] is a new class of low-density lipoprotein cholesterol-lowering drug that has recently been approved for the treatment of primary hypercholesterolemia or mixed dyslipidemia in adults, alone or in combination with other lipid-lowering therapies such as statins or ezetimibe. After administration, BemA is transformed to its CoA derivative by the enzyme very-long-chain acyl-CoA synthetase-1 (ACSVL1), which is primarily expressed in the liver [2]. The CoA derivative is the active form of the compound, which acts by inhibiting the enzyme ATP-citrate lyase (ACLY) [2]. ACLY catalyzes the conversion of citrate, generated in mitochondria from glucose, into acetyl-CoA. This reaction is one of the initial steps of cholesterol synthesis in the hepatocyte, preceding the statin target hydroxymethyl glutaryl CoA reductase, which is the rate-limiting enzyme of cholesterol biosynthesis [1]. However, acetyl-CoA is not only used in cholesterol, but also in fatty acid biosynthesis, after its conversion to malonyl-CoA in a reaction catalyzed by acetyl-CoA carboxylase (ACC) [3]. Therefore, ACLY is a key enzyme involved in both cholesterol and triglyceride (TG) metabolism.

Due to its role in fatty acid and TG synthesis, ACLY is one of the enzymes that controls hepatic lipid levels. The accumulation of TG in the liver (hepatic steatosis) is a hallmark of non-alcoholic fatty liver disease (NAFLD), which may evolve to steatohepatitis, cirrhosis, and hepatocellular carcinoma [4]. NAFLD is one of the most common hepatic diseases in industrialized countries, with a current global prevalence of 25%, that is expected to increase in the next decade [5]. However, there is a lack of specific pharmacological treatment for NAFLD, and its management is based on lifestyle changes (diet and exercise), which are difficult to sustain indefinitely [6]. Therefore, there is an urgent need for pharmacotherapies to treat NAFLD. Recently, we developed a model of hepatic steatosis without obesity and without inflammation in female rats [7,8]. To obtain this model, we used a high-fat diet without cholesterol, to avoid hepatic inflammation, and supplemented it with a 10% *w*/*v* fructose solution (HFHFr) to produce fatty liver due to an increase in de novo lipogenesis (DNL), associated with increased ACLY gene and protein expression [8].

The role of ACLY in NAFLD has been poorly studied, although it has been shown that hepatic DNL is upregulated in individuals suffering NAFLD [9]. In animal models, liver-specific deletion of ACLY led to conflicting results regarding hepatic lipid accumulation. Thus, in leptin receptor-deficient mice, knocking down hepatic ACLY expression protected against hepatic steatosis [10], whereas hepatic ACLY abrogation increased TG levels in the liver in wild-type mice fed either a low-fat or a high-fat diet [11].

We hypothesized that ACLY inhibition by BemA, leading to reduced fatty acid synthesis, could be a novel strategy to manage NAFLD. Under this scenario, we showed in a preliminary report [12] that BemA significantly reduced hepatic TG concentration in the HFHFr model. Using the same rats, here we confirm a marked anti-steatotic effect of BemA in this model by neutral lipid histological Oil Red O staining, and identify several mechanisms, besides the inhibition of ACLY, accounting for it: ketohexokinase (KHK) repression, patatin-like phospholipase domain-containing protein 3 (PNPLA3) induction, and peroxisome proliferator-activated receptor (PPAR) agonist activity.

## 2. Materials and Methods

### 2.1. Animals and Experimental Design

Two-month-old female Sprague Dawley rats weighing 178 ± 8 g (Envigo, Barcelona, Spain) were housed two per cage under conditions of constant humidity (40–60%) and temperature (20–24 °C), with a light/dark cycle of 12 h. Twenty-four rats were randomly assigned into three groups (n = 8 each) and fed the diets for 3 months: (i) the control group (CT) was fed a regular chow diet (2018 Teklad Global rodent diet, Envigo, Barcelona, Spain), with free access to water; (ii) the high-fat, high-fructose group (HFHFr) was fed a high-fat diet and had free access to a 10% *w*/*v* fructose solution; (iii) the bempedoic acid group (BemA) was fed the HFHFr diet and was treated orally with 30 mg/kg/day of BemA (MedChemTronica, Sollentuna, Sweden) by gavage during the third month of treatment. Solid food and liquid consumption were controlled three times a week, and body weight was recorded once a week. The composition of the high-fat diet (Teklad Custom Diet TD. 180456, Envigo, Madison, WI, USA) is described in Velázquez et al. [7]. The dose, length of treatment, and preparation of BemA dosing solution were based on previous studies performed in rats [13]. Briefly, a disodium salt aqueous solution of BemA was prepared, and carboxymethyl cellulose (CMC) and Tween-20 were added to make a solution containing 0.5% CMC and 0.025% Tween-20. CT and HFHFr groups were gavaged with the vehicle. In the CT and BemA groups, one rat was euthanized before the end of the experimental period, so the final n for these groups was 7. Procedures involving animals were carried out according to institutional guidelines established by the Bioethics Committee of the University of Barcelona (Autonomous Government of Catalonia Act 5/21 July 1995). All experiments performed on animals were approved by the Animal Experimentation Ethics Committee of the University of Barcelona (approval no. 10106).

### 2.2. Oral Glucose Tolerance Test

An oral glucose tolerance test (OGTT) was performed in the last week of the treatment as described previously [7]. Glucose levels were determined in blood samples using a handheld glucometer (Accutrend Plus System, Cobas, Roche Farma, Barcelona, Spain). Serum was obtained from blood samples collected at 0, 15, and 120 min, and insulin levels were measured using a rat insulin enzyme-linked immunosorbent assay (ELISA) kit (Millipore, Billerica, MA, USA).

### 2.3. Sample Preparation

At the end of the treatment, rats fasted for 2 h and blood samples were obtained from the tail vein to measure TG, cholesterol, and glucose levels, using an Accutrend Plus system glucometer (Cobas, Roche Farma, Barcelona, Spain). The rats were then immediately anesthetized with ketamine/xylazine (9 mg/40 µg per 100 g of body weight, respectively) and blood was collected into micro-tubes (Sarstedt AG & Co., Nümbrecht, Germany) through cardiac puncture and centrifuged at 10,000× *g* for 5 min at room temperature. Rats were euthanized by exsanguination, and the liver, subcutaneous, and perigonadal white adipose tissue (sWAT and pWAT, respectively) were dissected and weighed. Samples of brown adipose tissue (BAT) were also obtained. For histological studies, samples of the liver of each animal were fixed overnight in 10% neutral buffered formalin solution, or were embedded in OCT (Tissue-Tek^®^, Torrance, CA, USA), frozen quickly in liquid nitrogen, and stored at −80 °C. The remaining liver tissues were perfused, immediately frozen in liquid nitrogen, and stored at −80 °C until needed for biomolecular assays.

### 2.4. Serum Analytes

Serum adiponectin, insulin, and leptin concentrations were determined using specific ELISA kits (EZRADP-62K; EZRMI-13K; and mEZRL-83K, respectively) from Millipore (Billerica, MA, USA). The non-esterified free fatty acids (NEFA) colorimetric kit was from Bioo Scientific (Austin, TX, USA), and the kits for alanine (ALT) and aspartate aminotransferase (AST) kinetics were from SpinReact (Girona, Spain). The insulin sensitivity index (ISI) was calculated as 2/[insulin (nM) × blood glucose (µM) + 1].

### 2.5. Histological Studies

Frozen liver samples embedded in OCT were stained with Oil Red O (ORO, Sigma-Aldrich, St. Louis, MO, USA). Images were acquired with a Leica DMSL microscope equipped with a DP72 camera (Leica Microsistemas, Barcelona, Spain) and analyzed using ImageJ 1.49 software (National Institutes of Health, Bethesda, MD, USA). The area of positive ORO staining was calculated as the positively stained area per total area. All procedures were carried out in the Animal Histopathology Laboratory at the University of Barcelona.

### 2.6. RNA Extraction and Quantitative RT-PCR Analysis

Total RNA was extracted from the tissues (50~100 mg of each sample) using a phenol-based method according to the manufacturer’s instructions (TRIzol^®^ Reagent, Carlsbad, CA, USA). The cDNA synthesis was performed as a total RNA extraction using M-MLV reverse transcriptase (Invitrogen, Carlsbad, CA, USA). Reverse transcription was performed in the presence of random hexamers (Roche, Meylan, France) and dNTP (Sigma-Aldrich, St. Louis, MO, USA). Specific mRNA expression levels were detected by quantitative RT-PCR using Power SYBR^®^ Green PCR Master Mix (Applied Biosystems, Thermo Fisher Scientific, Waltham, MA, USA). Target gene expression (2^−ΔΔCt^) was normalized to endogenous β-actin expression. The primer sequences, Genbank TM number, and PCR product lengths are listed in Table S1.

### 2.7. Protein Extraction and Western Blot

Liver samples were homogenized in a lysis buffer using a Potter–Elvehjem homogenizer, and total protein and nuclear extracts were prepared as described previously [7]. To prepare protein extracts form adipose tissue, the samples were homogenized in a lysis buffer using a TissueLyser LT (Qiagen, Düsseldorf, Germany) at 50 Hz for 10 min, centrifuged at 13,000× *g* for 15 min at 4 °C, and the supernatant was obtained. Protein content in protein extracts was determined by the Bradford assay [14]. Western blots were performed using four samples per group, each sample pooled from two animals, as described previously [8]. To confirm the uniformity of protein loading, blots were incubated with anti-β-actin or anti-β-tubulin antibodies (Sigma-Aldrich, St. Louis, MO, USA), or with an anti-vinculin antibody (Santa Cruz Biotech, Dallas, TX, USA), as a control for total protein extracts, and with an anti-TBP antibody (AbCam, Cambridge, UK) for nuclear protein extracts. A list of antibodies used in Western blot analysis is shown in Table S2.

### 2.8. Liver Lipidomic Analysis

Liver homogenates were obtained, levels of fatty acid methyl esters from TG were determined by gas chromatography/electron ionization/mass spectrometry after TG isolation by solid-phase extraction, and levels of diacylglycerols (DAG), ceramides (Cer), and hexosylceramides (HexCer) were determined by liquid chromatography-tandem mass spectrometry (LC–MS/MS), as described previously [7].

### 2.9. PPAR Array and qPCR Validation

PPAR signaling transcript analyses were conducted in triplicate in liver samples using a personalized qPCR array (SignArrays 96^®^ system, Anygenes, Paris, France) for rats. Gene profiling was performed for 84 genes of interest, normalized by eight reference genes as described by the manufacturer. Data analysis was conducted using AnyGenes^®^ Excel analysis tools based on the 2^−ΔΔCt^ method by calculating fold changes for each gene as the difference in gene expression between BemA and HFHFr liver samples. The expression of selected genes was validated by RT-qPCR using liver samples from CT, HFHFr, and BemA groups.

### 2.10. PPAR Luciferase Assay

To identify whether BemA has PPAR agonist activity, commercial reporter assay kits for human PPARα, δ and γ, and for rat PPAR α and γ from Indigo Biosciences (State College, PA, USA) were used. These kits utilize proprietary non-human mammalian cells engineered to provide high-level of expression of these receptors. The reporter cells provided with the kit were resuspended in media supplied by the manufacturer, dispensed into 96-well assay plates, and incubated with different concentrations of BemA and positive control agonists. Luminescence was quantified using a Modulus microplate reader (Turner Biosystems, Sunnyvale, CA, USA). Plots of relative light units vs. Log [compound] were generated by GraphPad Prism Software version 9 (San Diego, CA, USA). EC50 and Emax values were calculated via non-linear curve fitting by using STATGRAPHICS Centurion software, version 18.1.12.

### 2.11. β-Oxidation Activity

Total fatty acid β-oxidation was determined by the method of Lazarow [15] using 30 µg of post-nuclear supernatant from the liver samples.

### 2.12. Statistical Analysis

Results are expressed as mean ± standard deviation (SD). Significant differences were established by one-way ANOVA and Tukey’s post-hoc test (GraphPad Software version 9, San Diego, CA, USA). When the SD of the groups was different according to the Brown–Forsythe test, the data were transformed into their logarithms and ANOVA was rerun, or the corresponding non-parametric test (Kruskal–Wallis) was applied. The OGTT curves for glucose and insulin were analyzed by two-way ANOVA. The level of statistical significance was set at *p* < 0.05.

## 3. Results

### 3.1. Treatment with Bempedoic Acid Reduces Body Weight and Adiposity, and Increases Liver Weight, without Affecting the Expression of Thermogenesis Markers in WAT

As shown in Table 1, BemA treatment reduced the increase in total calorie intake induced by the HFHFr diet and caused a decrease in the final body weight in comparison to the control and HFHFr groups. Moreover, pWAT and sWAT relative weights were significantly reduced in BemA-treated rats compared with the HFHFr group. In contrast, the liver weight/body weight ratio of BemA rats was more than double that of control and HFHFr rats.

On the other hand, BemA treatment did not reduce the hypertriglyceridemia induced by the HFHFr diet and slightly increased blood cholesterol concentrations compared to the HFHFr group. Serum NEFA levels were marginally increased (*p* = 0.07) in the BemA group compared with the control group. There were no significant differences in serum ALT and AST concentrations, nor in adiponectin levels, whereas leptin levels showed a significant decrease in the BemA group, in accordance with the reduction in WAT weight.

**Figure 1:**
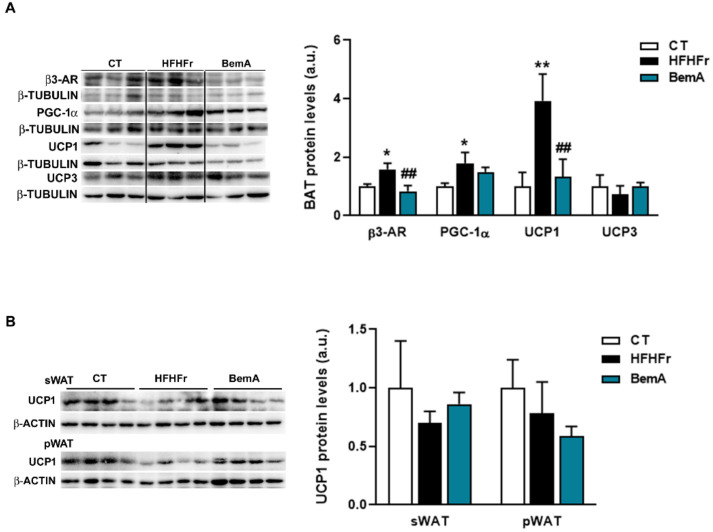
Bempedoic acid does not increase thermogenic activity in adipose tissue. Protein levels of β3-AR, PGC-1α2, UCP1, and UCP3 in brown adipose tissue (**A**) and of UCP1 in subcutaneous adipose tissue (sWAT), and perigonadal white adipose tissue (pWAT) (**B**) from control (CT), high-fat, high-fructose (HFHFr), and bempedoic acid (BemA)-treated rats. The results are expressed as the mean ± SD of four pooled samples for each condition, obtained by mixing equal amounts of tissue from two rats. On the left side of the figure, representative Western blot bands corresponding to the three different study groups are shown. a.u.: arbitrary units. Data were analyzed by one-way ANOVA, followed by Tukey’s post-hoc test. * *p* < 0.05, ** *p* < 0.01 compared to CT; ## *p* < 0.01 compared to HFHFr.

We next determined whether the decrease in body weight observed in BemA-treated rats was due to an increase in the thermogenic activity in adipose tissue. Protein levels of thermogenic markers β3-adrenergic receptor and uncoupling protein 1 (UCP1) in BAT samples of BemA rats were reduced compared to the HFHFr samples (Figure 1A). No significant differences were observed for UCP1 in sWAT or pWAT (Figure 1B). These results suggested non-increased thermogenic activity.

### 3.2. Rats Treated with Bempedoic Acid Reduce Their Fructose Ingestion and the Expression of Hepatic Ketohexokinase

**Figure 2:**
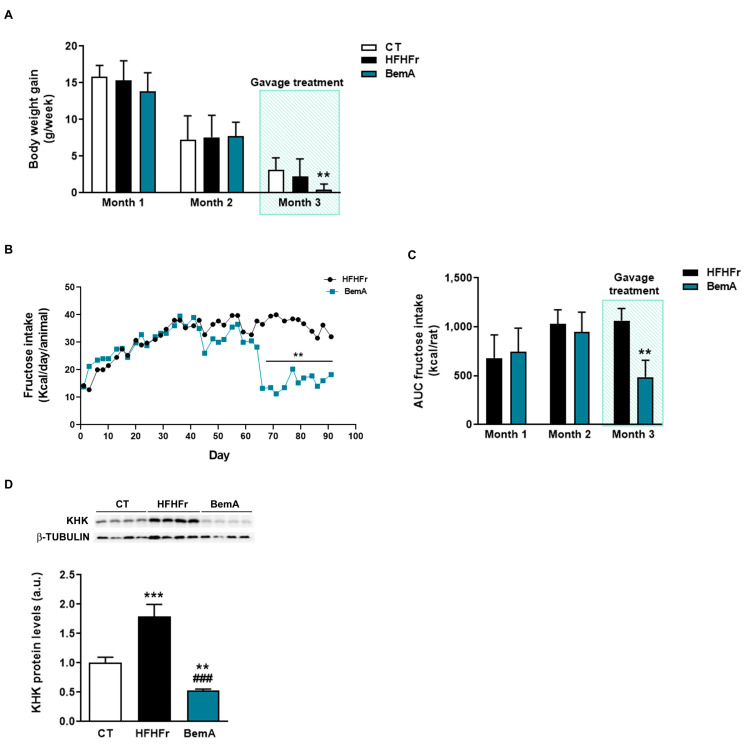
Bempedoic acid treatment reduces body weight gain, fructose intake, and KHK expression. (**A**) Body weight gain of control (CT), high-fat, high-fructose (HFHFr), and bempedoic acid (BemA)-treated rats during the three months of treatment. (**B**) Fructose intake of the rats from the HFHFr and BemA groups during treatment. (**C**) Area under the curve (AUC) of fructose intake of the rats from the HFHFr and BemA groups corresponding to the first two months and last month of treatment. (**D**) Protein expression of KHK in liver samples from CT, HFHFr, and BemA groups. In the upper part of the figure, representative Western blot bands corresponding to the three different study groups are shown. The results are expressed as the mean ± SD of 7–8 animals/group. For Western blot analysis, four pooled samples for each condition, obtained by mixing equal amounts of tissue from two rats, were used. a.u.: arbitrary units. Data were analyzed by one-way ANOVA, followed by Tukey’s post-hoc test. ** *p* < 0.01, *** *p* < 0.001 compared to CT; ### *p* < 0.001 compared to HFHFr.

In order to further explore the effects of BemA on final body weight, the rats’ weight gain was analyzed per month. As can be seen in Figure 2A, body weight gain was reduced in the BemA group only in the third month in relation to the control group. This reduction in body weight coincided with the start of BemA treatment and appeared in parallel with a significant reduction in fructose intake (Figure 2B,C). This suggests that BemA may have a specific effect of lowering fructose consumption, which could be related to an alteration in fructose metabolism. It has been shown that mice lacking KHK, the first enzyme in the hepatic metabolism of fructose, also reduced their fructose ingestion [16]. Interestingly, our results show that KHK expression, which was significantly increased by the HFHFr diet, was reduced by BemA treatment below control levels (Figure 2D).

### 3.3. Bempedoic Acid Does Not Alter Insulin Sensitivity in High-Fat, High-Fructose Rats

**Figure 3:**
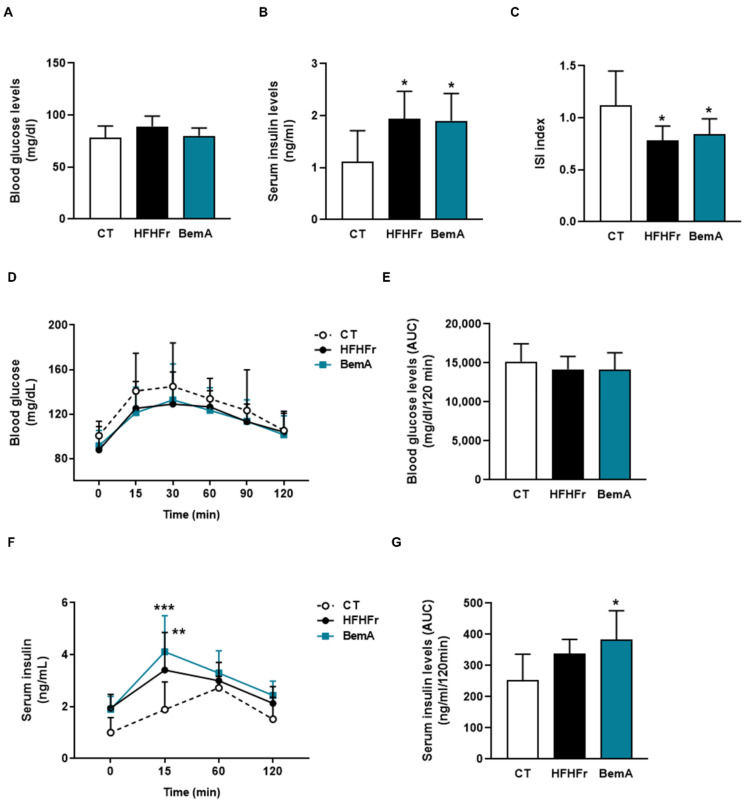
Bempedoic acid treatment does not correct the hyperinsulinemia induced by HFHFr. (**A**) Blood glucose and (**B**) serum insulin levels in control (CT), high-fat, high-fructose (HFHFr), and bempedoic acid (BemA)-treated rats at the end of the treatment. (**C**) Insulin sensitivity index (ISI) calculated as 2/[insulin (nM) X blood glucose (µM) + 1] in CT, HFHFr, and BemA rats. (**D**) Blood glucose levels and (**F**) serum insulin levels at different times after the glucose challenge in the oral glucose tolerance test, and the corresponding areas under the curves calculated from these values (**E**,**G**). The results are expressed as the mean ± SD of 7–8 animals/group. Data were analyzed by one-way ANOVA, followed by Tukey’s post-hoc test or by two-way ANOVA (**D**,**F**). * *p* < 0.05, ** *p* < 0.01, *** *p* < 0.001 compared to CT.

Next, we evaluated BemA effects on glucose metabolism. As previously described [7,8], the HFHFr diet did not modify basal glucose levels, but increased the serum insulin concentration, thus reducing the ISI; these alterations were not reversed by BemA treatment (Figure 3A–C).

Similarly, the results of the oral glucose tolerance test showed no differences in glucose levels and the corresponding areas under the curve (Figure 3D,E), whereas insulin concentration after 15 min of the glucose challenge and the insulin AUC were increased in the HFHFr group, without BemA being able to reverse this trend (Figure 3F,G).

### 3.4. Bempedoic Acid Reduces Hepatic TG Accumulation Induced by the High-Fat, High-Fructose Diet and Significantly Alters Hepatic Lipid Metabolism

**Figure 4:**
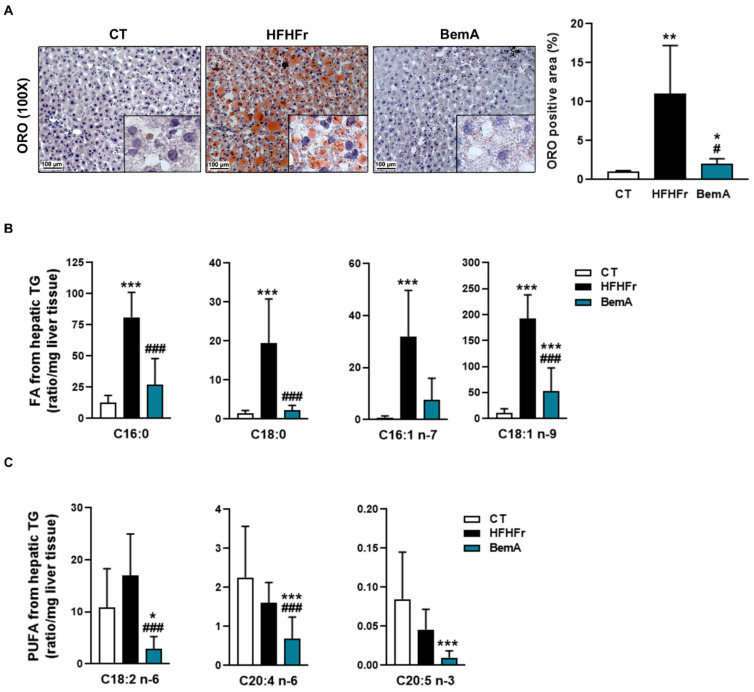
Bempedoic acid reduces liver steatosis and the amount of fatty acids from hepatic triglycerides. (**A**) Images of Oil Red O-stained sections, and quantification of the positive stained area, in livers from control (CT), high-fat, high-fructose (HFHFr), and bempedoic acid (BemA)-treated rats. Amount of saturated and monounsaturated (**B**), and polyunsaturated fatty acids (PUFA) (**C**) in liver triglycerides from CT, HFHFr, and BemA groups. The results are expressed as the mean ± SD of 7–8 animals/group. Data were analyzed by one-way ANOVA, followed by Tukey’s post-hoc * *p* < 0.05, ** *p* < 0.01, *** *p* < 0.001 compared to CT; # *p* < 0.05, ### *p* < 0.001 compared to HFHFr.

We previously reported that treatment of HFHFr rats with BemA reduced the amount of hepatic TG by 56% [12]. This effect was also observed when we analyzed the accumulation of liver fat using ORO-stained histological samples. As shown in Figure 4A, the ORO-stained positive area was increased around 10-fold in samples from HFHFr rats, and BemA administration drastically reduced it almost to control levels.

**Figure 5:**
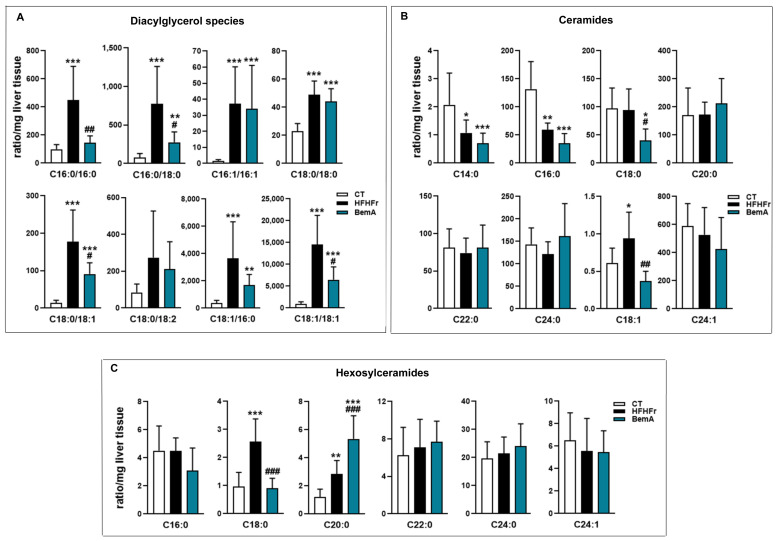
Bempedoic acid modifies the lipidomic signature induced by the HFHFr diet in the liver. Bar plots showing the levels of (**A**) diacylglycerols, (**B**) ceramides, and (**C**) hexosylceramides in rat liver homogenates from control (CT), high-fat, high-fructose (HFHFr), and bempedoic acid (BemA) groups. Data were analyzed by one-way ANOVA, followed by Tukey’s post-hoc * *p* < 0.05, ** *p* < 0.01, *** *p* < 0.001 compared to CT; # *p* < 0.05, ## *p* < 0.01 ### *p* < 0.001 compared to HFHFr.

We further examined the levels of the different fatty acid species present in hepatic TG (Figure 4B). BemA treatment abolished the increase in the levels of C16:0 (palmitic acid), C18:0 (stearic acid), C16:1 n-7 (palmitoleic acid), and C18:1 n-9 (oleic acid) induced by the HFHFr diet. In addition, BemA decreased the amount of several polyunsaturated fatty acids (C18:2 n-6, C20:4, n-6, and C20:5 n-3) that were not significantly modified by the HFHFr diet (Figure 4C). Similarly, the levels of the most abundant diacylglycerol (DAG) species in the rats’ liver were increased by the HFHFr diet, and treatment with BemA blunted these increases (Figure 5A). On the contrary, diet exerted minor effects on hepatic ceramide (Cer) levels; the most remarkable effects were the significant decreases in Cer C16:0, Cer C18:0, and Cer C18:1 in the livers of BemA-treated rats (Figure 5B). Regarding the hexosylceramides (HexCer), both HexCer C18:0 and C20:0 were significantly increased by the HFHFr diet, and BemA treatment reduced the amount of the former and further increased the amount of the latter (Figure 5C).

**Figure 6:**
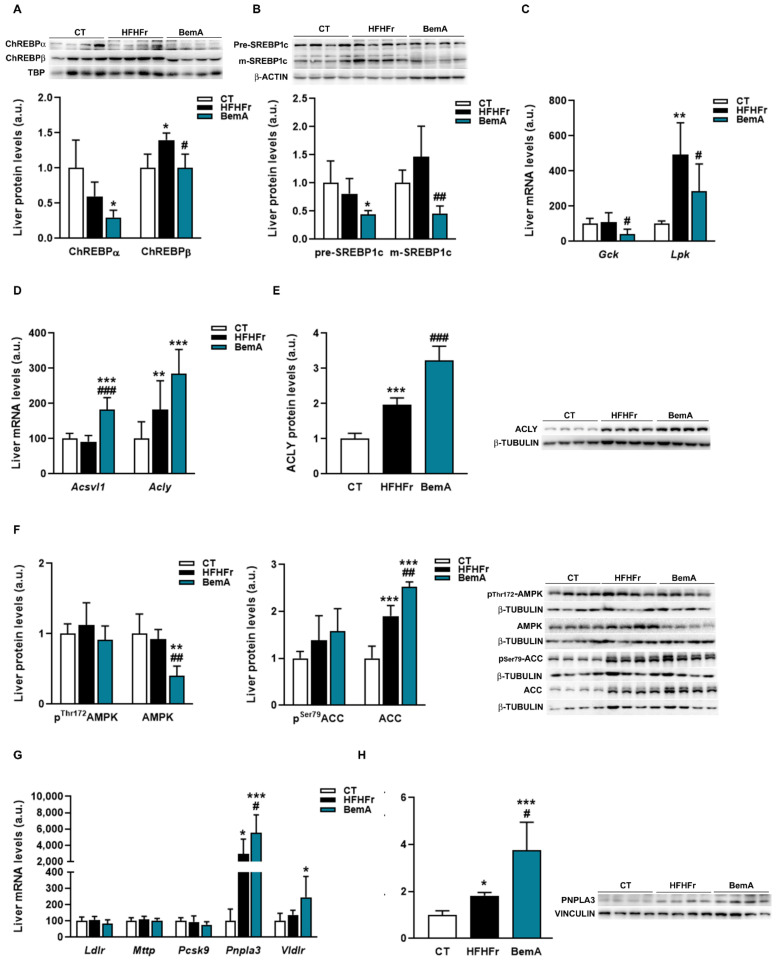
Effects of bempedoic acid on the expression of proteins controlling de novo lipogenesis and triglyceride trafficking. (**A**) Protein levels of ChREBP α and β, in nuclear extracts prepared from livers of control (CT), high-fat, high-fructose (HFHFr), and bempedoic acid (BemA)-treated rats. (**B**) Protein levels of precursor (pre) and mature (m) forms of SREBP1c in livers from CT, HFHFr, and BemA rats. In the upper part of the figures, representative Western blot bands corresponding to the three different study groups are shown. The mRNA levels of *Gck* and *Lpk* (**C**), *Acsvl1* and *Acly* (**D**) in liver samples from CT, HFHFr, and BemA groups. (**E**) Protein levels of ACLY in livers from CT, HFHFr, and BemA-treated rats. On the right side of the figure, representative Western blot bands corresponding to the three study groups are shown. (**F**) Levels of phospho^Thr172^ and total AMPK, and of phospho^Ser79^ and total ACC in livers from CT, HFHFr, and BemA rats; on the right side of the figure, representative Western blot bands corresponding to the three study groups are shown. (**G**) The mRNA levels of *Ldlr*, *Mttp*, *Pcsk9*, *Pnpla3,* and *Vldlr* in liver samples from CT, HFHFr, and BemA groups. (**H**) Protein levels of PNPLA3 in the livers from CT, HFHFr, and BemA-treated rats. On the right side of the figure, representative Western blot bands corresponding to the three different study groups are shown. The results are expressed as the mean ± SD of 7–8 animals/group (mRNA), or as the mean ± SD of four pooled samples for each condition, obtained by mixing equal amounts of tissue from two rats (Western blot analysis). a.u.: arbitrary units. Data were analyzed by one-way ANOVA, followed by Tukey’s post-hoc * *p* < 0.05, ** *p* < 0.01, *** *p* < 0.001 compared to CT; # *p* < 0.05, ## *p* < 0.01 ### *p* < 0.001 compared to HFHFr.

The marked reduction in hepatic steatosis caused by BemA treatment led us to examine the expression of several molecules that control hepatic DNL and TG trafficking. As shown in Figure 6A, the protein levels of both isoforms of carbohydrate response element binding protein (ChREBP)-⍺ and -β were significantly reduced in nuclear extracts from the livers of BemA-treated rats. Similarly, the amounts of the precursor and mature forms of sterol response element binding protein (SREBP)1c were reduced by BemA treatment (Figure 6B). Consequently, the mRNA expression of liver pyruvate kinase (*Lpk*) and glucokinase (*Gck*), controlled by ChREBPβ and SREBP1c, respectively, were also reduced in the liver of BemA rats (Figure 6C). On the other hand, BemA treatment increased the expression of very long chain acyl-CoA synthetase 1 (*Acsvl1*), as well as the expression and protein levels of ACLY (Figure 6D,E). Although BemA has previously been referred to as an AMP-dependent kinase (AMPK) activator [13], in our model, BemA did not increase liver phospho^Thr172^ AMPK, nor phospho^Ser79^ acetyl-CoA carboxylase (ACC) levels (Figure 6F), suggesting a lack of a BemA-related increase in liver AMPK activity in our experimental conditions.

Regarding the proteins involved in TG transport that we analyzed, the mRNA and protein levels of PNPLA3, which were increased by the HFHFr diet, were further enhanced by BemA treatment (Figure 6G,H). In addition, BemA treatment increased the hepatic expression of the very low-density lipoprotein receptor (*Vldlr*).

### 3.5. Bempedoic Acid Behaves as an Agonist of Human and Rat PPAR α/γ

**Figure 7:**
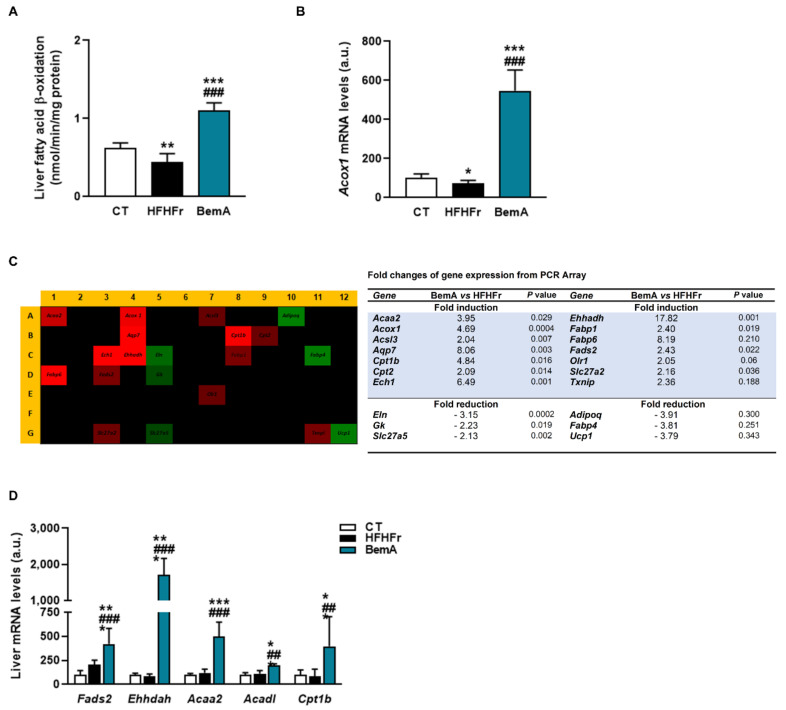
Bempedoic acid increases fatty acid β-oxidation in the liver. (**A**) β-oxidation activity in liver samples from control (CT), high-fat, high-fructose (HFHFr), and bempedoic acid (BemA) groups. (**B**) The mRNA levels of Acox in liver samples from CT, HFHFr, and BemA groups. (**C**) Heat map of PCR array showing differential expression of 84 PPAR-responsive genes in liver samples. The heat map represents fold regulation expression between HFHFr and BemA liver samples. Genes with higher expression in BemA samples compared to HFHFr are depicted in red, and genes with lower expression are depicted in green. On the right side of the figure, a table shows the name of the genes induced or repressed more than two-fold, and the *p* value obtained after the statistical analysis using Student’s *t*-test. (**D**) Validation by RT-PCR of genes related to fatty acid peroxisomal (*Fadss2*, *Ehhadh*) and mitochondrial (*Acaa2*, *Acadl,* and *Cpt1b*) β-oxidation in CT, HFHFr, and BemA liver samples. The results are expressed as the mean ± SD of 7–8 animals/group. a.u.: arbitrary units. Data were analyzed by one-way ANOVA, followed by Tukey’s post-hoc * *p* < 0.05, ** *p* < 0.01, *** *p* < 0.001 compared to CT; ## *p* < 0.01 ### *p* < 0.001 compared to HFHFr.

The increase in liver weight ratio (Table 1) and the apparent hepatocyte hypertrophy observed in our preliminary report [12] suggested a peroxisomal proliferator-like profile for BemA. Indeed, BemA treatment significantly increased hepatic β-oxidation activity and the expression of the rate-limiting enzyme of the peroxisomal β-oxidation pathway acyl-CoA oxidase (*Acox1*) (Figure 7A,B). This is consistent with the reduction in the amount of fatty acids in the liver, particularly of very long polyunsaturated fatty acids, the preferential substrate of peroxisomal fatty acid β-oxidation. Given that Acox1 is under the transcriptional control of PPARα, we next used a qPCR panel to determine the expression of 84 PPAR-regulated genes in liver samples from the HFHFr and BemA groups. Our results showed that BemA induced a more than two-fold change in the expression of 20 of these PPAR-responsive genes, of which 14 were upregulated and 6 were downregulated (Figure 7C). The significant increase in the hepatic mRNA levels of several enzymes related to fatty acid peroxisomal (*Fadss2*, *Ehhadh*) and mitochondrial (*Acaa2*, *Acadl*, and *Cpt1b*) β-oxidation after BemA treatment was validated by RT-PCR (Figure 7D).

**Figure 8:**
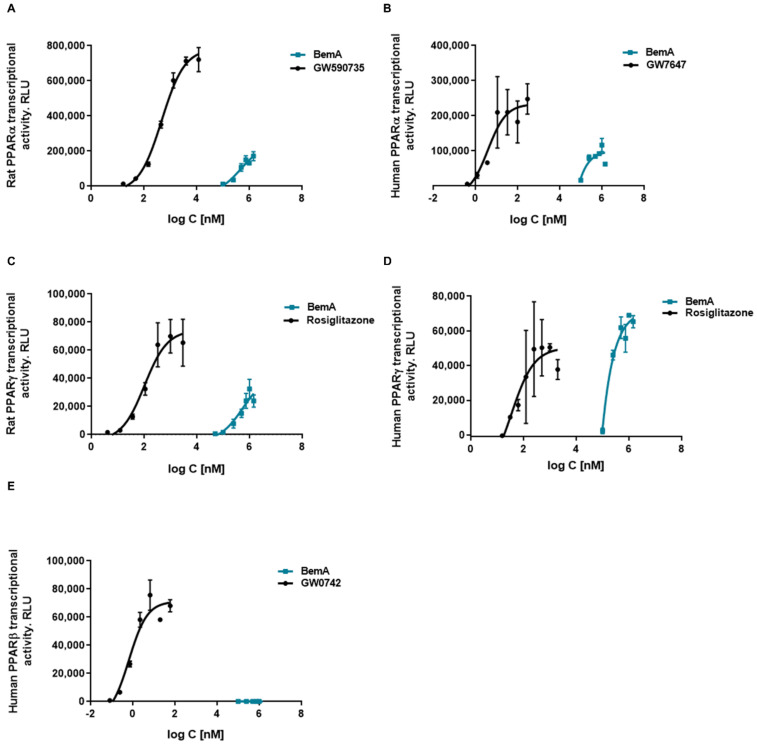
Concentration response curves of bempedoic acid in a cell-based reporter gene assay. Effects of bempedoic acid (BemA) on rat PPARα (**A**), human PPARα (**B**), rat PPARγ (**C**), human PPARγ, (**D**) and human PPARβ (**E**) luciferase reporter gene expression. GW590735, GW7647, rosiglitazone, and GW0742 were used as positive controls, respectively. RLU = relative light units. The average of 2–3 measurements and standard deviations are shown.

These results, together with the chemical structure of BemA (an ω-dicarboxylic fatty acid), suggest that this compound could be acting as a PPAR agonist. Previously, we found that the binding of PPARα to a peroxisome proliferator response element (PPRE) was increased in liver samples from BemA rats compared to HFHFr liver samples [12]. To verify that BemA induced the transcriptional activity of PPAR, we used luciferase reporter assay kits based on mammalian cells expressing several isoforms of rat and human PPAR. The results of these assays showed that the transcriptional activity of human and rat PPARα and γ was increased by BemA treatment, while human PPARβ remained unaffected (Figure 8). BemA behaves as a low potency rat and human PPARα agonist, shown by higher EC50 and lower efficacy values than the classical agonists GW590735 and GW7647 (Table 2). Regarding rat and human PPARγ, BemA also showed low potency compared to the reference agonist rosiglitazone, but its efficacy was similar (Table 2).

## 4. Discussion

NAFLD, and specifically the initial phase of the pathology spectrum, simple hepatic steatosis, represents an unmet medical need. This stage has usually been regarded as a benign, non-progressive condition, but several recent studies show that it has similar potential to NASH to evolve to fibrosis [17,18]. Thus, the management of simple steatosis is of great importance to prevent its evolution towards more advanced disease. Previously, we established a model of this initial stage of NAFLD, fatty liver without inflammation, by feeding female rats a high-fat diet enriched in palmitic acid and stearic acid, devoid of cholesterol, and supplemented with 10% *w*/*v* fructose in drinking water (HFHFr) [7,8]. By using this model, in the present work we show that BemA treatment alleviates hepatic steatosis by several mechanisms, including reduced KHK expression, PNPLA3 induction, and PPAR⍺ activation. In addition to these mechanisms, which have not been described previously, our results indicate that BemA inhibits ACLY activity, which was indirectly shown by a compensatory increase in the mRNA and protein levels of the enzyme, but does not activate AMPK phosphorylation in our model.

KHK, expressed mainly in the liver and the intestine, is an essential enzyme for fructose metabolism, so fructose cannot be metabolized in humans with loss-of-function mutations or in KHK-knockout mice [19]. In our rat model, fat consumption alone leads only to a mild state of steatosis, whereas the addition of liquid fructose drives the marked accumulation of intrahepatic TG due to enhanced DNL [8]. Therefore, the inhibition of hepatic KHK expression by BemA is key to reducing intrahepatic fat levels in the HFHFr rats, as it may lead to the inability to properly metabolize fructose in the liver, preventing fructose-induced DNL. Accordingly, the expression of transcription factors controlling hepatic lipogenesis (ChREBP and SREBP), the levels of palmitic, palmitoleic, and oleic acid in hepatic TG (markers of DNL), and most of the DAG species analyzed, were reduced by BemA treatment. In turn, KHK inhibition may be mediated, at least in part, by the reduction in the expression of the constitutively active ChREBPβ isoform, as it is a direct target of this transcription factor [20]. Nevertheless, the fact that the expression of KHK in BemA rats was 0.5-fold lower than control values points to an additional direct effect of BemA, independent from ChREBPβ activity.

In our study, solid food consumption was similar between the HFHFr and BemA groups (8.1 ± 1.0 and 7.9 ± 0.8 g/rat/day, respectively), but BemA treatment caused a drastic decrease in fructose drinking. Despite this reduction was not causing dehydration (as the amount of liquid consumed in ml/rat/day was not different from the CT group, data not shown), the reduction in fructose drinking significantly reduced the energy intake in the BemA rats compared to the HFHFr group. While we cannot rule out that intestinal discomfort might be the cause of reduced fructose intake, we did not observe diarrhea nor any other signs of toxicity in BemA-treated rats. Instead, we attribute the lower fructose intake to the reduction in KHK expression caused by BemA. It has been reported that mice with a global deletion of KHK reduce their liquid fructose consumption compared to wild-type mice, and are protected from weight gain and fat accretion, as well as from fatty liver [16], similar to our BemA-treated rats. Furthermore, a recent study showed that KHK deletion had different effects depending on the tissue: specific hepatic KHK deletion did not reduce fructose drinking, but protected from fatty liver, whereas the deletion of KHK only in the jejunum led to reduced fructose intake, but hepatic steatosis was promoted due to reduced intestinal fructose metabolism and increased entry of fructose into the liver [21]. Thus, although we could not determine the expression of intestinal KHK, we can infer from our results (reduced fructose drinking accompanied by reduced hepatic steatosis) that BemA may reduce KHK expression not only in the liver, but also in the intestine.

A second mechanism that may underlie the anti-steatosis effect of BemA is the increase in PNPLA3 expression. PNPLA3, also known as adiponutrin, acts as a TG hydrolase and acyltransferase [22], and its expression is regulated by ChREBP and SREBP [23]. While the physiological function of PNPLA3 has not been clearly elucidated, a polymorphism in this gene driving the loss of its hydrolase activity (the I148M variant) is strongly associated with NAFLD [24]. Furthermore, although the studies in PNPLA3 knockout or transgenic mice that overexpressed PNPLA3 led to conflicting results [25,26,27], the loss-of-function I148M variant has been shown to promote hepatic steatosis [28,29]. The observed increase in PNPLA3 expression after BemA treatment may therefore contribute to the reduction of liver fat content, independently from ChREBP/SREBP. Two recent reports showed that overexpression of mutant forms of PNPLA3 leading to a loss of function caused a specific lipidomic signature in the liver of mice, characterized by the accumulation of very long chain polyunsaturated fatty acids (PUFA) in TG [30] and ceramides [31]. Our results are consistent with these reports showing a role of PNPLA3 in lipid remodeling, as BemA treatment significantly reduced the amount of C20:4 n-6 and C20:5 n-3 in hepatic TG and the levels of Cer C16:0, Cer C18:0, and Cer C18:1 in the liver.

Interestingly, despite a clear beneficial effect of BemA on liver lipids, plasma cholesterol and TG levels were not reduced by the treatment. PNPLA3 overexpression in *db*/*db* mice has been shown to increase plasma TG levels through increased very low-density lipoprotein (VLDL) production [26], whereas the I148M variant reduces hepatic VLDL secretion [32] and leads to lower serum TG levels, at least in obese individuals [33]. According to these reports, the lack of a hypotriglyceridemic effect of BemA observed in our study could be related to the increase in PNPLA3 expression. Moreover, despite the fact that we did not determine VLDL production, the regulatory role of PNPLA3 on this process points to the increased secretion of these lipoproteins. As cholesterol is mainly transported in VLDL in rats, this effect would counteract the cholesterol-lowering effect of BemA, explaining the increase in plasma cholesterol levels observed in our model.

The third mechanism that contributes to the steatosis-reducing effect of BemA is PPAR activation. PPARs are a group of transcription factors that regulate the expression of target genes involved in, among other functions, lipid homeostasis [34]. In our model, BemA exhibited peroxisome proliferator-like properties, such as the promotion of hepatomegaly and hepatocyte hypertrophy, which are characteristic effects of PPARα agonists in rodents [35]. Moreover, we observed increased β-oxidation and significant changes in the expression of a panel of PPAR-regulated genes in the liver of our BemA-treated rats. These results prompted us to examine the ability of BemA to act as a PPAR agonist. Indeed, luciferase gene reporter assays showed that BemA activates rat and human PPAR⍺, although with lower potency and efficacy compared to the reference agonists GW590735 and GW7647. Moreover, BemA also behaves as a low potency agonist of rat and human PPARγ, achieving a similar Emax to the classical PPARγ full agonist rosiglitazone. The chemical structure of BemA, a ω-dicarboxylic fatty acid, is similar to the endogenous PPAR agonists, and may confer the ability to activate these receptors. However, the relevance of these findings in our in vivo model differs for each PPAR subtype. Thus, despite behaving as a weak PPAR⍺ activator in the reporter gene assays, BemA induced the expression of several PPAR⍺-responsive genes, including *Vldlr*, as well as genes involved in both peroxisomal (*Acox*, *Ehhadh*, *Fads2*), and mitochondrial (*Acaa2*, *Acadl*) fatty acid β-oxidation. The stimulation of hepatic lipid catabolism clearly contributed to the reduction of hepatic steatosis in our model. In contrast, the typical PPARγ-related effects, such as increased adipogenesis and adiponectin levels were not observed in our in vivo model. Moreover, the expression of PPARγ target genes in the pWAT of BemA-treated rats (*CD36*, *Ap2*, *Lpl*, and *Pepck*) was not increased (data not shown). Given that PPARγ is mainly expressed in adipose tissue, whereas the highest PPAR⍺ expression is observed in the liver [36], one possibility is that BemA exerts a liver-specific effect and does not activate PPARγ in adipose tissue. In support of this hypothesis, the induction of hepatic *Acsvl1* means that BemA is mainly converted to a CoA derivative when it enters the liver, and probably does not reach peripheral tissues such as adipose tissue in meaningful concentrations for the activation of PPARγ.

One limitation of our study is that BemA effects were investigated in an animal model, and the translation to patients with NAFLD may not be straightforward. However, in our model, the induction of DNL by fructose feeding is the main driver of hepatic steatosis [8] and, similarly, the contribution of DNL to the establishment of hepatic steatosis in NAFLD patients is high, reaching almost 40% [37]. In addition, excessive fructose consumption, leading to higher DNL, is one of the main dietary contributors to fatty liver and other metabolic disturbances [38]. Therefore, the effects of BemA observed in our model may also be relevant in humans suffering NAFLD. In this sense, reduced DNL through KHK inhibition has recently emerged as a novel therapeutic approach to reduce hepatic steatosis [39]. On the other hand, increased PNPLA3 expression by BemA may lead to different outcomes in humans depending on the PNPLA3 genotype. Thus, in carriers of the loss-of-function PNPLA3 I148M variant, the induction of PNPLA3 may not have a clear anti-steatotic effect. On the contrary, the increase in PNPLA3 expression in individuals carrying the wild-type active protein would be effective at reducing liver fat by stimulating both lipolysis and the secretion of TG-rich lipoproteins to the blood. However, this last effect would counteract the reduction of plasma TG levels caused by the inhibitory effect of BemA on fatty acid synthesis. This could explain the lack of a hypotriglyceridemic effect of BemA in clinical studies [40]. Finally, regarding the effect of BemA as a PPARα agonist; although PPARα is less abundant in human liver than in rodents [41], its expression in the liver of NASH patients is negatively correlated with the severity of steatosis and inflammation, and histological improvement is strongly associated with an increase in hepatic PPARα expression [42]. Moreover, PPARα activation does not cause a proliferative response in human hepatocytes, and the long-term clinical use of hypolipidemic PPARα agonists is not associated with hepatic hypertrophy and liver cancer [43].

In summary, our findings reveal that BemA is able to reduce hepatic steatosis in a rat model resembling the initial phases of human NAFLD. In humans, drugs that exclusively target PPAR⍺, such as fenofibrate, do not reduce liver fat content [44,45]. The fact that BemA has multiple mechanisms of action, including not only PPARα agonism, but also KHK inhibition and PNPLA3 induction, makes it an attractive candidate for NAFLD treatment.

## Figures and Tables

**Table 1:** Zoometric parameters and blood, serum, and liver analytes.

	CT	HFHFr	BemA
Total calorie intake (kcal/90 days/rat)	3648 ± 133	5914 ± 178 ***	5219 ± 420 *** ^#^
Body weight (g)	262 ± 12	269 ± 14	244 ± 9 * ^##^
Liver weight (g)	7.9 ± 0.6	9.5 ± 1.5	15.8 ± 1.4 *** ^#^
Liver weight/BW (%)	3.0 ± 0.3	3.6 ± 0.6	6.4 ± 0.5 *** ^#^
pWAT/BW (%)	0.9 ± 0.1	0.9 ± 0.2	0.6 ± 0.3 ^#^
sWAT/BW (%)	1.2 ± 0.3	1.5 ± 0.5	1.0 ± 0.1 ^#^
Blood TG (mg/dL)	118 ± 15	197 ± 65 *	231 ± 71 **
Blood Chol (mg/dL)	160 ± 3	158 ± 5	181 ± 21 ^#^
NEFA (mmol/L)	0.2 ± 0.1	0.3 ± 0.1	0.4 ± 0.1
ALT (UI/L)	26 ± 6	25 ± 6	32 ± 8
AST (UI/L)	43 ± 6	37 ± 13	40 ± 8
Adiponectin (µg/mL)	32 ± 5	36 ± 15	26 ± 7
Leptin (ng/mL)	3.3 ± 0.8	4.0 ± 1.2	1.3 ± 0.3 *** ^###^

ALT: alanine aminotransferase; AST: aspartate aminotransferase; BW: body weight; Chol: cholesterol; NEFA: non-esterified free fatty acids; pWAT: perigonadal white adipose tissue; sWAT: subcutaneous white adipose tissue; TG: triglyceride. Values are expressed as means from 7–8 animals ± SD, except for total calorie intake, which are means from four cages ± SD. * *p* < 0.05, ** *p* < 0.01, *** *p* < 0.001 vs. CT: # *p* < 0.05, ## *p* < 0.01, ### *p* < 0.001 vs. HFHFr.

**Table 2:** EC50 and Emax values for bempedoic acid and standard PPAR agonists.

PPAR Isoform	Compound	EC50	Emax
Rat PPARα	BemA	781 (0–1737) µM	261675 (107,548–415,802)
	GW590735	561 (392–730) nM	761689 (704,593–818,785)
Rat PPARγ	BemA	876 (0–2803) µM	181745 (0–381,356)
	Rosiglitazone	78 (31–125) nM	177321 (153,254–201,388)
Human PPARα	BemA	168 (0–670) µM	107342 (29,995–184,688)
	GW7647	5.3 (0–11.1) nM	236160 (181,649–290,670)
Human PPARγ	BemA	332 (0–962) µM	87195 (32,506–141,883)
	Rosiglitazone	76 (0–176) nM	51652 (35,820–67,485)
Human PPARβ	BemA	-	-
	GW0742	0.98 (0–2.10) nM	72221 (54,980–89,461)

EC50: Half maximal effective concentration; Emax: Maximal effect. Values and 95% confidence intervals were calculated from the concentration-response curves via non-linear curve fitting by using the STATGRAPHICS Centurion software, version 18.1.12.

## Data Availability

The data presented in this study is contained within the article. Individual values are available from the corresponding authors upon reasonable request.

## Supplementary Materials

The following supporting information can be downloaded at: https://www.mdpi.com/article/10.3390/biomedicines10071517/s1, Table S1: Genes, GenBank Number, SYBR Green primer sequences, and PCR products; Table S2: Antibodies used in Western blot analysis.
