# Supplementary material for: KHK, PNPLA3 and PPAR as Novel Targets for the Anti-Steatotic Action of Bempedoic Acid
Source: Biomedicines. 2022 Jun 27;10(7):1517. doi: 10.3390/biomedicines10071517 (PMC9312949; doi:10.3390/biomedicines10071517)
Supplement: Supplementary file 1 [file biomedicines-10-01517-s001.zip › biomedicines-1778316-supplementary.pdf]

Table S1. Genes, GenBank Number, SYBR Green primer sequences and PCR products.

Gene	GenBank TM	Primer Sequences	PCR Product
Acly	NM_016987.2	F: 5′-CCCTTCGTCCCCCACAGT-3′<br>R: 5′-TCTCCTTCCCGGGTAGCAT-3′	65 bp
Acaa2	NM_130433.1	F: 5′-CCCTGCTATCATGGGGATCG-3′<br>R: 5′-GAGCAAATGCTTCATTCACGTCT-3′	110 bp
Acadl	NM_012819.2	F: 5′-CCCGATTGCAAAAGCCTACG-3′<br>R: 5′-ACTGACGATCTGTCTTGCGA-3′	97 bp
Acox	NM_017340.2	F: 5′-GTGAGGCGCCAGTCTGAAA -3′<br>R: 5′- ACTGCTGGGTTTGAAAATCCA-3′	70 bp
Acsl1	NM_012820.2	F: 5′-TGTGGGGTGGAAATCATCGG-3′<br>R: 5′- TTGGGGTTGCCTGTAGTTCC-3′	131 bp
Acsvl1	NM_031736.2	F: 5′- ATGGCGTGCCTCAACTACAA-3′<br>R: 5′-GGGTTGGAAGAACCTCCTCG-3′	124 bp
Chrebpα	NM_001393706.1	F: 5′-AGCATCGATCCGACACTCAC-3′<br>R: 5′-TGTTCAGCCGAATCTTGTCC-3′	130 bp
Chrebpβ	NM_133552.2	F: 5′-TGTTCAGCCGAATCTTGTCC -3′<br>R: 5′-TGTTCAGCCGAATCTTGTCC -3′	125 bp
Cpt1b	NM_013200.2	F: 5′- CAGTGTGCCAGCCACAATTC -3′<br>R: 5′- TAGGCTTCGTCATCCAGCAA -3′	66 bp
FadS2	NM_031344.2	F: 5′- TCATCGACCGCAGGTCTAC -3′<br>R: 5′- CGGAAGGCATCCGTAGCAT -3′	105 bp
Gck	NM_001270849.1	F: 5′-ACTGCCGAGATGATGAAGCA-3′<br>R: 5′-GCCCTTGGTCCAATTGAGGA-3′	70 bp
Ldlr	NM_175762.3	F: 5′- AGACCCAGAGCCATCGTAGT -3′<br>R: 5′- TTGATCTTGGCAGGTGTCCC -3′	77 bp
L-pk	NM_012624.3	F: 5′- GAACATTGCACGACTCAACTTCT-3′<br>R: 5′- CGGATGTTGGCGATGGAT-3′	69 bp
Mttp	NM_001107727.1	F: 5′-TCTCTGCTGACCCGCATTTT-3′<br>R: 5′-GCTACCGGATCTCATCGGAC-3′	122 bp
Pcsk9	NM_199253.2	F: 5′-ACATGTCACAGAGTGGGACG-3′<br>R: 5′-TCCCGGTTCAGCATCATAGC-3′	73 bp
Pnpla3	NM_001282324.1	F: 5′- AGTCTCCCTCTCGATCACATCA -3′<br>R: 5′- TGTTGAAGAACGGGTGGAGG -3′	94 bp
Tbp	NM_001004198.1	F: 5′-TGGGATTGTACCACAGCTCCA-3′<br>R: 5′-CTCATGATGACTGCAGCAAACC-3′	132 bp
Vldlr	NM_013155.2	F: 5′-CCAGGAACAGGACTGCAGAG-3′<br>R: 5′-GCCACCGTTATTGACCAAGC-3′	82 bp

Table S2. Antibodies used in western blot analysis.

Antibody	Reference Number
ACLY	Cell Signaling (#4332)
AMPK	Cell Signaling (#2532)
p-AMPK (Thr^{172})	Cell Signaling (#2535)
ACC	Cell Signaling (#3662)
p-ACC (Ser^{79})	Cell Signaling (#3661)
β3-AR	Santa Cruz (sc-515763)
β-ACTIN	Sigma Aldrich (A5441)
β-TUBULIN	Sigma-Aldrich (T4026)
ChREBP (P-13)	Santa Cruz (sc-21189)
KHK	Santa Cruz (sc-50029)

PGC-1α	Cayman (101707)
PNPLA3	Invitrogen (PA5-117066)
SREBP1c	Santa Cruz (sc-366)
UCP1 (A-6)	Santa Cruz (sc-518024)
VINCULIN	Santa Cruz (sc-73614)
